# Persistent Inhibition of ABL Tyrosine Kinase Causes Enhanced Apoptotic Response to TRAIL and Disrupts the Pro-Apoptotic Effect of Chloroquine 

**DOI:** 10.1371/journal.pone.0077495

**Published:** 2013-10-11

**Authors:** Priya Sridevi, May K. Nhiayi, Ryan L. Setten, Jean Y. J. Wang

**Affiliations:** Moores Cancer Center, Division of Hematology-Oncology, Department of Medicine, School of Medicine, University of California San Diego, La Jolla, California, United States of America; Innsbruck Medical University, Austria

## Abstract

TNF-Related Apoptosis Inducing Ligand (TRAIL) binds to and activates death receptors to stimulate caspase-8 and apoptosis with higher efficiency in cancer than normal cells but the development of apoptosis resistance has limited its clinical efficacy. We found that stable, but not transient knockdown of the ABL tyrosine kinase enhanced the apoptotic response to TRAIL. Re-expression of Abl, but not its nuclear import- or kinase-defective mutant, in the ABL-knockdown cells re-established apoptosis suppression. TRAIL is known to stimulate caspase-8 ubiquitination (Ub-C8), which can facilitate caspase-8 activation or degradation by the lysosomes. In the ABL-knockdown cells, we found a higher basal level of Ub-C8 that was not further increased by lysosomal inhibition. Re-expression of Abl in the ABL-knockdown cells reduced the basal Ub-C8, correlating with apoptosis suppression. We found that lysosomal inhibition by chloroquine (CQ) could also enhance TRAIL-induced apoptosis. However, this pro-apoptotic effect of CQ was lost in the ABL-knockdown cells but restored by Abl re-expression. Interestingly, kinase inhibition at the time of TRAIL stimulation was not sufficient to enhance apoptosis. Instead, persistent treatment for several days with imatinib, an ABL kinase inhibitor, was required to cause the enhanced and the CQ-insensitive apoptotic response to TRAIL. Together, these results show that persistent loss of nuclear ABL tyrosine kinase function can sensitize cells to TRAIL and suggest that long-term exposure to the FDA-approved ABL kinase inhibitors may potentiate apoptotic response to TRAIL-based cancer therapy.

## Introduction

Tumor necrosis factor-related apoptosis-inducing ligand (TRAIL) and its death-domain receptors DR4 and DR5 have been well documented for their role in the activation of extrinsic apoptosis [1]. The pro-apoptotic TRAIL action follows the paradigm of Fas-ligand induced and caspase-8-dependent apoptosis, and it preferentially induces apoptosis in cancerous and immortalized non-cancerous cells [2]. This cancer cell-specific apoptotic response to TRAIL has yet to be successfully translated into a clinically efficacious therapeutic outcome because of the rapid onset of TRAIL resistance, which is attributed but not limited to TRAIL receptor mutation [3], increased expression of TRAIL decoy receptors DcR1 and DcR2 [4], decreased caspase-8 expression due to promoter hypermethylation [5], increased expression of c-FLIP that inhibits caspase-8 cleavage [6], increase expression of Bcl2 [7] or XIAP [7], and activation of the NF-kB pathway [8]. 

A variety of agents, including genotoxins, chromatin modifiers, proteasome inhibitors, kinase inhibitors and inhibitors of anti-apoptotic proteins have been shown to sensitize cancer cells to TRAIL-induced apoptosis [9]. In particular, chemotherapeutic drugs such as cisplatin, doxorubicin, 5-fluorouracil and gemcitabine have been shown to sensitize colon, pancreas, prostate and breast cancer cells to TRAIL [10-14]. Cisplatin and doxorubicin are known to activate the nuclear ABL tyrosine kinase, which stimulates the p53-family of transcription factors to activate mitochondria-dependent intrinsic apoptosis [15-22]. Recent studies have shown that TRAIL can activate the DNA damage response (DDR) as a result of caspase-dependent DNA fragmentation, and this DDR response contributes to TRAIL-induced apoptosis [23,24]. This caspase-induced DDR may explain the recently reported activation of the ABL-JNK-apoptosis pathway by TRAIL [25]. Besides the DDR, caspase-dependent cleavage/degradation of RB also leads to activation of ABL and p73 to enhance TNF and TRAIL induced apoptosis [15]. Together, these published results show that the nuclear ABL tyrosine kinase can enhance cell killing by TRAIL through activation of intrinsic apoptosis. 

The ubiquitously expressed ABL tyrosine kinase plays an essential role in embryonic development as Abl-knockout mice exhibit an array of defects and die in utero or soon after birth [26]. The N-terminal region of ABL contains the SH3, SH2 and kinase domains that can adopt an auto-inhibited intramolecular assembly, which is disrupted in the constitutively activated v-Abl and BCR-ABL oncogenic kinases [15,27,28]. The C-terminal region of ABL contains three nuclear localization signals (NLS), one nuclear export signal (NES) and an F-actin binding domain that regulate the subcellular localization of this protein kinase [29,30]. In proliferating cells, ABL shuttles between the cytoplasm and the nucleus, and this dynamic equilibrium is subjected to regulation by cell adhesion and DNA damage [17,19,31,32]. In the cytoplasm, ABL is activated by a variety of extracellular signals including growth factors, cytokines, antigens, matrix attachment and microbial infection to regulate F-actin-dependent biological processes such as membrane ruffling, cell migration and vesicle trafficking [15,31,33-37]. In the nucleus, ABL is activated by DNA damage to regulate transcription, DNA repair and apoptosis [18,38]. As discussed above, nuclear ABL kinase can stimulate the pro-apoptotic function of the p53-family of transcription factors to activate DNA damage-induced intrinsic apoptosis [16,17,19,39]. The tyrosine kinase activity of ABL is inhibited by imatinib, nilotinib and dasatinib, which are used in the clinic to treat chronic myelogenous leukemia (CML) caused by the oncogenic BCR-ABL tyrosine kinase [40-43]. CML patients have been treated with daily dosing of imatinib for more than a decade, showing that the persistent inhibition of ABL kinase activity does not cause life-threatening side effects during adult life [40-42]. 

We undertook this study to investigate the role of nuclear ABL tyrosine kinase in genotoxin-induced TRAIL sensitization with the expectation that nuclear ABL would stimulate TRAIL-induced apoptosis through the DDR pathway. However, we came upon the unexpected finding that loss of nuclear ABL tyrosine kinase function could cause cells to become more sensitive to TRAIL-induced extrinsic apoptosis. This “anti-apoptotic” effect of nuclear ABL kinase on extrinsic apoptosis differs from its “pro-apoptotic” effect on intrinsic apoptosis. The “pro-apoptotic” function of ABL in DDR is blocked by kinase inhibition at the time of exposure to genotoxins [19,44,45]. However, the “anti-apoptotic” effect of ABL is only disrupted after persistent inhibition of its kinase activity for several days.

## Materials and Methods

### Reagents

Recombinant human TRAIL was from Millipore (EMD Millipore, Billerica, MA) and doxorubicin and chloroquine were from Sigma (Sigma-Aldrich, St. Louis, MO). Bafilomycin A1 and IETD-AFC were from Enzo Life Sciences (Enzo Life Sciences Inc., Farmingdale, NY). DEVD-AMC, Annexin V apoptosis detection kit and FADD, RIP1, PUMA, Bcl-X and p62 antibodies were from BD Pharmingen (BD Biosciences, San Jose, CA). Abl-pY245, PARP1, caspase-8, cleaved caspase-3, Bid, FLIP, NF-kB, IKB-α, survivin, XIAP, Ubiquitin-P4D1 and horseradish peroxidase (HRP)-conjugated secondary antibodies were from Cell Signaling Technology (Danvers, MA). DR-4, DR-5 and goat anti-caspase-8 antibodies were from Santa Cruz (Santa Cruz Biotechnology Inc., Santa Cruz, CA) and GAPDH antibody was from Millipore (EMD Millipore, Billerica, MA). LC3 antibody was from MBL (MBL International Corporation, Woburn, MA). Protein A/G beads magnetic beads and chemiluminescence western blot developing kit were from Pierce (Pierce Biotechnology, Rockford, IL). 

### Cell Culture and Drug Treatment

HT29, MCF-7, MCF-10A and HCT116 cell lines were from ATCC. Except for MCF-10A, cells were maintained in DMEM (Cellgro) supplemented with 10% FBS (HyClone) and antibiotics (100 Units/ml penicillin and 100 µg/ml streptomycin from Cellgro). MCF-10A cells were cultured in DMEM-F12 (Invitrogen) containing 5% horse serum (Invitrogen), 20ng/ml EGF (Peprotech), 0.5 mg/ml Hydrocortisone (Sigma), 100 ng/ml Cholera toxin (Sigma), 10 µg/ml Insulin (Sigma) and antibiotics. For drug treatments 2x10^6^ cells were seeded per well of a 12-well plate overnight. Specified concentrations of recombinant human TRAIL (in 0.1% BSA in PBS), doxorubicin, chloroquine, imatinib, zVADfmk, dasatinib, nilotinib or Bafilomycin A1, were added to the cells and harvested at the indicated time points. Vehicle controls were H_2_O for doxorubicin, chloroquine, imatinib, and DMSO for zVADfmk, dasatinib, nilotinib, Bafilomycin A1.

### ABL Knockdown and Rescue

ABL was stably knocked down in HT29, HCT116, MCF-7 or MCF10A cells using lentivirus particles carrying an shRNA expression cassette to generate an ABL siRNA with the sequence GCAGTCATGAAAGAGATCAAACTC (vector backbone pLKO.1-puro from Sigma). Polybrene (EMD-Millipore, Billerica, MA) at 8 µg/ml concentration was added to aid infection. Stably infected cells were selected for puromycin-resistance and checked for ABL knockdown by immunoblotting. Cell infected with pLKO.1.PURO empty vector were used as vector control. ABL knockdown HT29 cells were further infected with retrovirus (MSCV) expressing the full length (WT), kinase defective (KD), nuclear import mutant (µNLS) or nuclear export mutant (µNES) murine Abl (type IV) which is not recognized by the ABL-shRNA. The above rescue cell lines were selected for hygromycin resistance and verified by immunoblotting and immunofluorescence. 

### Immunoblotting

After drug treatment, cells were washed with ice-cold PBS and lysed in RIPA buffer (25 mM Tris-HCl pH 7.4, 1 mM EDTA, 150 mM NaCl, 0.1% SDS, 1% NP-40, 1% Sodium Deoxycholate, 1 mM phenylmethylsulfonyl fluoride, 10 mM sodium orthovanadate, 10 mM beta-glycerophosphate, 50 mM sodium fluoride, 10 mM tetrasodium pyrophosphate and protease inhibitor cocktail), sonicated and centrifuged at 12,000 rpm for 10 mins. The supernatant was quantified using Biorad Lowry protein assay reagent and 30-50 µg lysate was used per lane of SDS-PAGE. The gels were transferred and western blotting was done following standard protocols.

### Immunofluorescence

Cells seeded in 24-well plates on cover slips were fixed with 4% paraformaldehyde for 20 mins, permeabilized with 0.3% Triton x-100 for 20 mins and blocked with 5% bovine serum albumin for 30 mins. The coverslips were incubated with anti-Abl (8E9) antibody (1:100) for 2 hrs at 37°C. Secondary antibody incubation was done using ALEXA fluor-488 tagged chicken anti-mouse (1:500) for an hour. Nuclei were counterstained with DAPI (4',6-diamidino-2-phenylindole dihydrochloride). The cells were photographed at 400X magnification using Zeiss LSM Confocal Microscopy and Image SPOT – image analysis software.

### IETDase and DEVDase Assay

At the end of drug treatment, cells were harvested by scraping on ice, washed in ice-cold PBS and were lysed in CHAPS buffer (10 mM HEPES-KOH (pH 7.5), 10 mM KCl, 1.5 mM MgCl2, 1 mM EDTA, 1 mM EGTA). The lysates were sonicated and incubated with 100 µM Ac-IETD-AFC or 40 µg/ml Ac-DEVD-AMC at 37°C. Fluorometric detection of AMC and AFC was performed in triplicates by excitation at 360 nm/emission at 460 nm (AMC) or excitation at 400 nm/emission at 500 nm (AFC) at 30 mins, 60 mins and 120 mins of reaction.

### Annexin V Binding Assay

Cells were harvested after drug treatment by trypsinization and were washed in PBS. Annexin V positive cells were analyzed using Annexin V cell labeling kit (R & D Systems) according to manufacturer’s instructions and FACS was done using BD FACSCalibur. 

### SubG1 Assay

After drug treatment, cells were harvested by trypsinization, washed in PBS and fixed overnight in 70% ethanol, in -20°C. Cells were then incubated with the DNA staining buffer containing 0.1% sodium citrate, 0.3% Triton-X100, 0.01% propidium iodide and 0.002% RNAse A, for 15 min in the dark. DNA fragmentation was analyzed by FACS assay.

### Immunoprecipitation

1X10^7^ cells were treated with the indicated drugs and after treatment, cells were harvested by scraping on ice. Cells were washed with ice-cold PBS and were lysed in DISC immunoprecipitation (IP) buffer containing 0.2% NP-40, 20 mM Tris-HCl (pH 7.4), 150 mM NaCl, 10% glycerol and protease inhibitor cocktail. The lysates were incubated on ice for an hr after which they were spun down twice at 4°C, for 10 mins at 12000 rpm. Cell supernatants were quantified by Lowry assay and were incubated overnight with goat anti-caspase-8 antibody (Santa Cruz Antibodies, Santa Cruz, CA). Magnetic protein AG beads (10 µl) from Pierce Biotechnology Inc. (Rockford, IL) were then added to pull down the caspase-8 complexes. The beads were washed in DISC IP buffer 3 times and proteins were eluted from the beads by heating with SDS sample buffer. The eluted proteins were run on the gel and western blotting was done using standard protocols. For detection of polyubiquitinated caspase-8, 1X10^7^ cells were lysed under reducing conditions in 1.0 ml of a modified RIPA buffer containing 1.0% SDS. The lysate was sonicated, centrifuged and boiled for 10 min at 95°C, following which immunoprecipitation was done with goat anti-caspase-8 antibody. Caspase-8 immunoprecipitates were pulled down as described above and ubiquitinated caspase-8 was detected using anti-ubiquitin P4D1 antibody.

### Clonogenic Survival Assay

2X10^6^ cells in 12-well plates were treated with the indicated drugs for 16 hr. In case of prolonged high dose TRAIL treatment, after 16 hr of treatment with 100 ng/ml TRAIL, the media along with the dead cells was removed and replaced with fresh TRAIL containing media every 24 hr for a total of 48 hr. At the end of treatment, cells were trypsinized, washed, resuspended in 1.5 ml media and 250 µl were plated in 4 wells of a 48-well plate. After 48 hr, when the vehicle-treated wells became confluent, the cells were stained with crystal violet solution (0.2% crystal violet in 4% PFA) for 10 min at room temperature. The wells were washed in PBS 3 times and air-dried. The wells were incubated with 250 µl SDS solution containing 0.5% w/v SDS in 50% v/v ethanol [46] and 0.5 M Tris-HCl pH - 7.8 for 10 min at room temperature to extract the crystal violet dye. The supernatant was mixed and absorbance at 586 nm was read using a spectrophotometer. Percent cell viability was determined by comparing the colorimetric units of the vehicle-treated vs TRAIL treated cells.

### Statistical Analysis

Statistical significance of data was determined using student t-test program in Microsoft Excel. p values less than 0.05 were considered to be statistically significant.

## Results

### Stable knockdown of ABL enhances TRAIL-induced apoptosis

**Figure 1:**
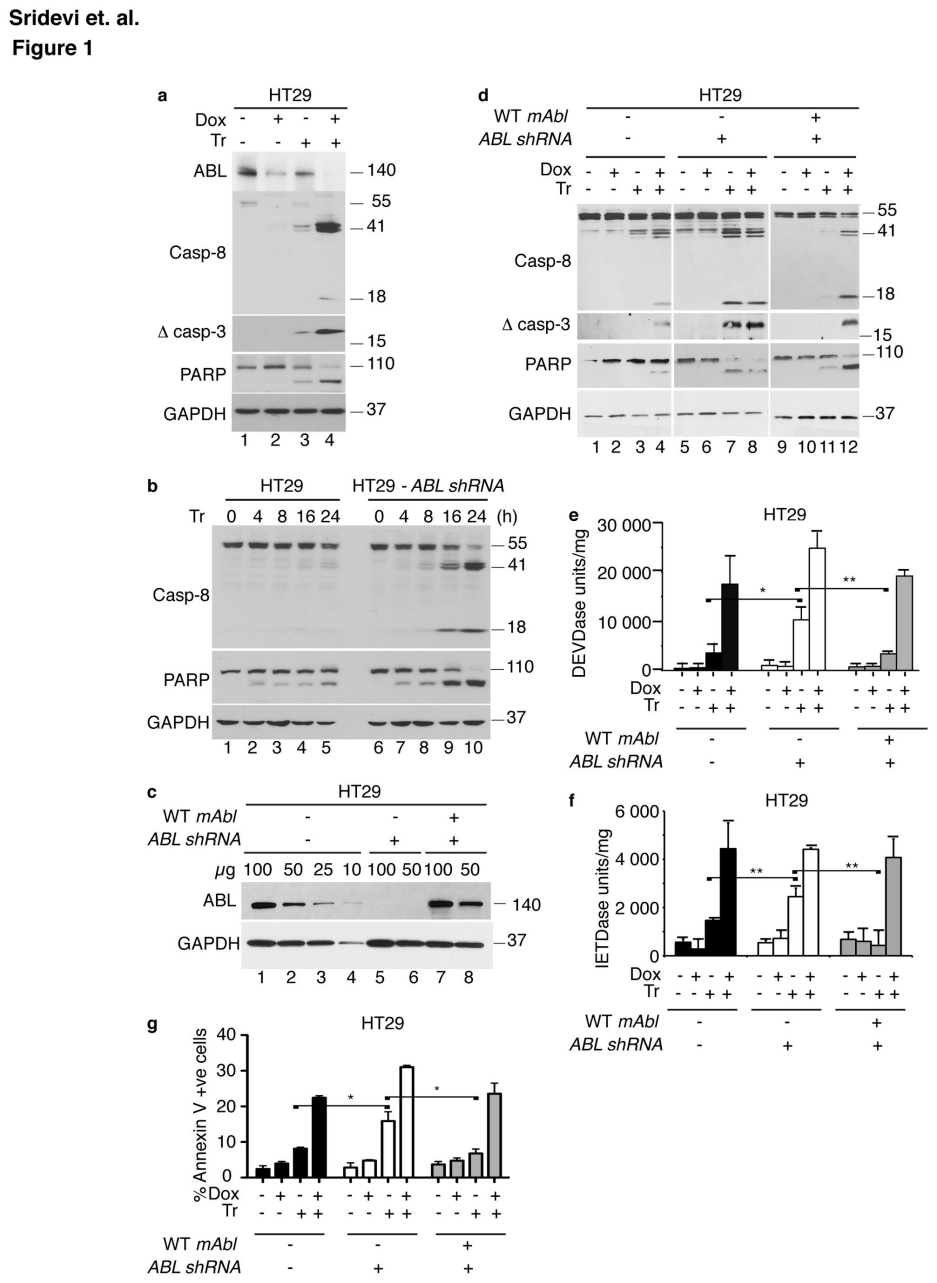
Stable knockdown of ABL enhances TRAIL-induced apoptosis. a. Doxorubicin and TRAIL caused ABL down-regulation. HT29 cells pretreated with doxorubicin (Dox, 2.0 µg/ml, 24 hr) or not were treated with TRAIL (Tr - 200 ng/ml, 8 hr) or not and whole cell lysates were immunoblotted with the indicated antibodies. b. ABL knockdown stimulated TRAIL-induced caspase activation. The indicated HT29 cells were treated with 200 ng/ml TRAIL. Whole cell lysates were collected at the indicated time points and blotted with the indicated antibodies. c. ABL levels in HT29 cells without (-) or with (+) the ABL-shRNA or the wild type (WT) mouse type-IV Abl (mAbl). The indicated amounts of whole cell lysates (µg) were loaded in each lane. d. Re-expression of mAbl in ABL-knockdown cells suppressed TRAIL-induced caspase activation. Caspase and PARP1 cleavage in the indicated HT29 cells treated with doxorubicin (2 µg/ml) and/or TRAIL (200 ng/ml) for 8 hrs. e.,f. DEVDase and IETDase activity in the indicated HT29 cells treated as in (d) with doxorubicin and/or TRAIL. ABL-knockdown significantly enhanced TRAIL-induced DEVDase (e) and IETDase (f). Reconstitution of mAbl expression significantly reduced DEVDase (e) and IETDase (f). Values shown are mean and standard deviation from three independent experiments each with duplicate measurements. * = p < 0.05; ** = p <0.01. g. Annexin V staining of the indicated HT29 cells treated as in (d) with doxorubicin and/or TRAIL. ABL-kockdown significantly enhanced TRAIL-induced Annexin V positivity. Reconstitution of mAbl expression significantly reduced Annexin V positivity. Values shown are mean and standard deviations from three independent experiments each with duplicate measurements. * = p < 0.05.

TRAIL-induced activation of caspase-8 can be enhanced by doxorubicin (Dox) in the colon cancer cell line HT29 [11]. We found that treatment with Dox or TRAIL caused a reduction in ABL protein levels and their effects were additive when cells were treated with Dox+TRAIL (Figure 1a). To determine if this reduction of ABL might affect TRAIL-induced apoptosis, we stably expressed an ABL-shRNA in HT29 cells to knockdown its expression (Figure 1c). When treated with TRAIL, the ABL-knockdown (ABL-k.d.) cells showed increased cleavage of caspase-8 and PARP1 relative to the parental HT29 cells (Figure 1b). Expression of the shRNA-vector or an irrelevant shRNA that did not target ABL had no effect on TRAIL-induced caspase activation (data not shown). We then expressed the wild type murine type-IV Abl (mAbl), which could not be silenced by the ABL-shRNA, in the ABL-k.d. cells (Figure 1c). We measured TRAIL-induced cleavage of caspase-8, caspase-3, PARP1 (Figure 1d), DEVDase (Figure 1e), IETDase (Figure 1f) and Annexin V staining (Figure 1g) in the parental, the ABL-k.d., and the mAbl-reconstituted HT29 cells. With each assay, we found a higher apoptotic response to TRAIL in the ABL-k.d. than the parental cells, and that each elevated response was suppressed by the re-expression of mAbl (Figure 1d-g). We also found that the apoptosis-enhancing effect of Dox was reduced but not abolished by the knockdown of ABL and restored by the re-expression of mAbl (Figure 1d-g). The knockdown of ABL also enhanced TRAIL-induced caspase activation in HCT116, MCF7 and MCF10A cells (Figure S1a-f). Together, these results showed that ABL-knockdown could enhance TRAIL-induced apoptosis. 

### Inhibition of TRAIL-induced apoptosis requires ABL kinase activity and nuclear localization

**Figure 2:**
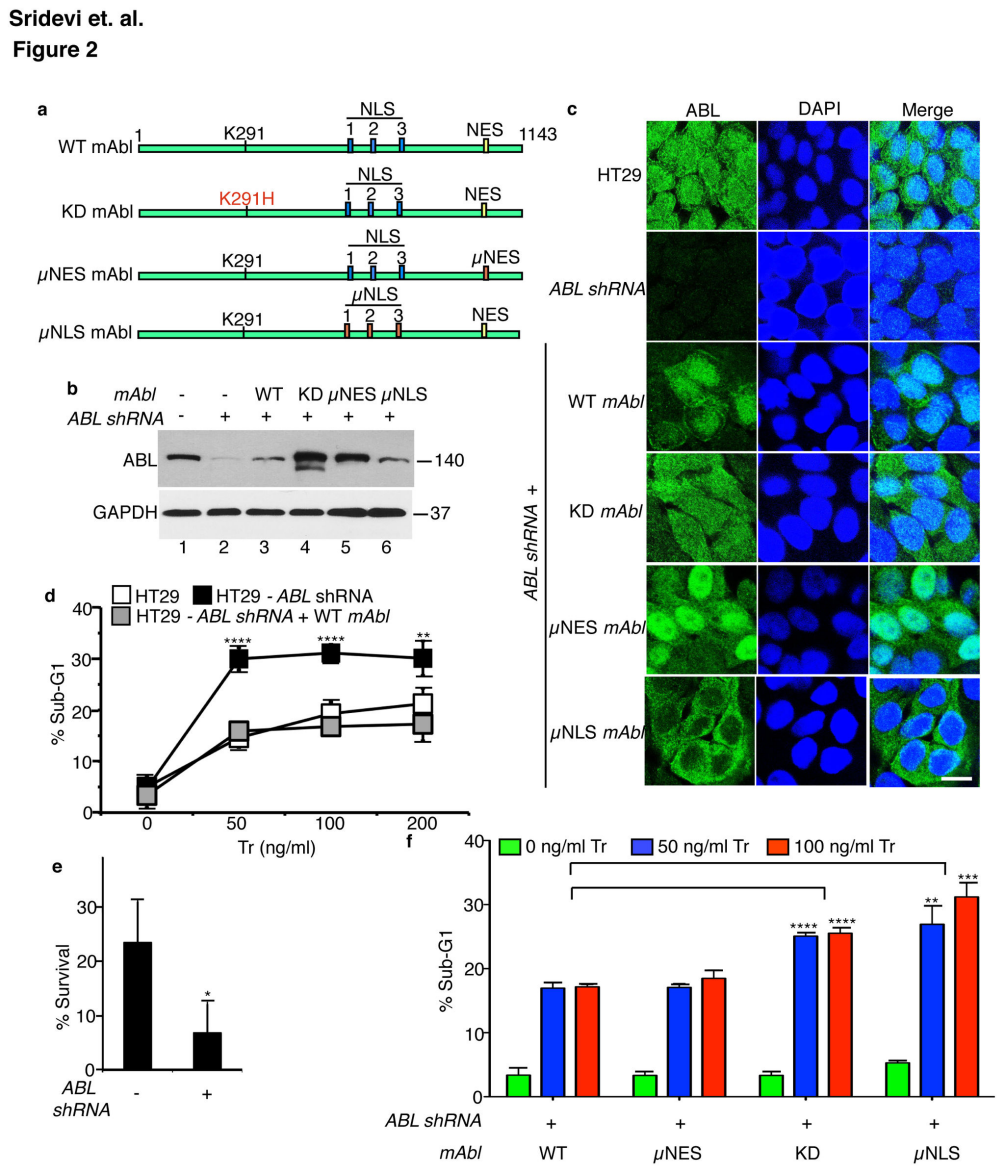
Inhibition of TRAIL-induced apoptosis requires ABL kinase activity and nuclear localization. a. Schematic representation of the Type VI mAbl cDNA constructs used in reconstitution experiments. WT, wild type; KD, kinase defective (K291H); µNLS, nuclear localization signals mutated (11 K/R substitutions with Q); µNES, nuclear export signal mutated (L1064A). b. Levels of endogenous ABL and mAbl proteins in whole cell lysates of the indicated HT29 cells. c. Immunofluorescence staining of the indicated HT29 cells with anti-ABL (green) and DAPI (blue). Scale bar - 20 µm. d. ABL knockdown enhanced, and reconstitution with mAbl, suppressed TRAIL-induced sub-G1. Cells treated with the indicated doses of TRAIL were collected at 16 hr and analyzed for sub-G1 by FACS. Values shown are mean and standard deviation from five independent experiments. **** = p < 0.00005 and ** = p < 0.005. e. ABL knockdown reduced survival. Cells were treated with 100 ng/ml Tr daily for three days, replated in media without Tr for another 3-days and cell survival measured by crystal violet assay (see Materials and Methods). Values shown are mean and standard deviation from three independent experiments each with four repeats. * p = 0.03. f. Inhibition of TRAIL-induced apoptosis requires mAbl kinase activity and nuclear import. Cells were treated with the indicated doses of Tr as in (d). Values shown are mean and standard deviations from 5 independent experiments. ** = p < 0.005, *** = p < 0.0005, **** = p < 0.00005.

To gain insights on how ABL might inhibit the apoptotic response to TRAIL, we re-expressed the kinase-defective (KD, Lys291His) [47], the nuclear import-defective (µNLS) [30], or the nuclear export-defective (µNES) [29] mAbl mutants in the ABL-k.d. cells (Figure 2a-c). The KD-mAbl was expressed at higher levels than the WT-mAbl (Figure 2b), possibly because the kinase-defective Abl was more stable [48]. The WT- and KD-mAbl proteins were distributed in the cytoplasm and the nucleus (Figure 2c). By contrast, the nuclear import-defective (µNLS) mAbl was localized exclusively to the cytoplasm whereas the nuclear export-defective (µNES) mAbl was predominantly nuclear (Figure 2c). As shown in Figure 2d, TRAIL-induced sub-G1 levels were consistently higher in the ABL-k.d. cells. This enhanced apoptotic response translated into a reduced clonogenic survival of the ABL-k.d. cells (Figure 2e). Re-expression of WT-mAbl in the ABL-k.d. cells reduced the sub-G1 response to that of the parental cells (Figure 2f). However, re-expression of KD-mAbl in the ABL-k.d. cells did not significantly alter the sub-G1 response (Figure 2f). The µNES-mAbl, which is mostly localized to the nucleus, also reduced the sub-G1 response (Figure 2f). By contrast, the µNLS-mAbl, which is only found in the cytoplasm, did not reduce the sub-G1 response (Figure 2f). Taken together, these results show that suppression of TRAIL-induced apoptosis required the ABL tyrosine kinase activity and its nuclear localization. 

### Effects of ABL-knockdown and mAbl-reconstitution and lysosomal inhibitors on ubiquitinated caspase-8

Previous studies have linked TRAIL resistance to several mechanisms, including the downregulation of its receptors, the upregulation of FLIP or other anti-apoptotic proteins [3-8]. We examined the levels of TRAIL receptors (DR4 and DR5), FADD, caspase-8, FLIP, RIP1, NF-kB-p65 (RelA), IkB-alpha, Bid, PUMA-alpha, Bcl-xL, XIAP, and survivin in the parental and the ABL-k.d. HT29 cells but did not find any changes that could readily explain the enhanced apoptotic response (Figure S2). We also found that TRAIL-induced co-immunoprecipitation of RIP1 with caspase-8 and IkB-alpha phosphorylation to occur in both the parental and the ABL-k.d. cells (Figure S2), suggesting that TRAIL-induced multi-protein complex formation and signaling were not affected in ABL-k.d. cells.

**Figure 3:**
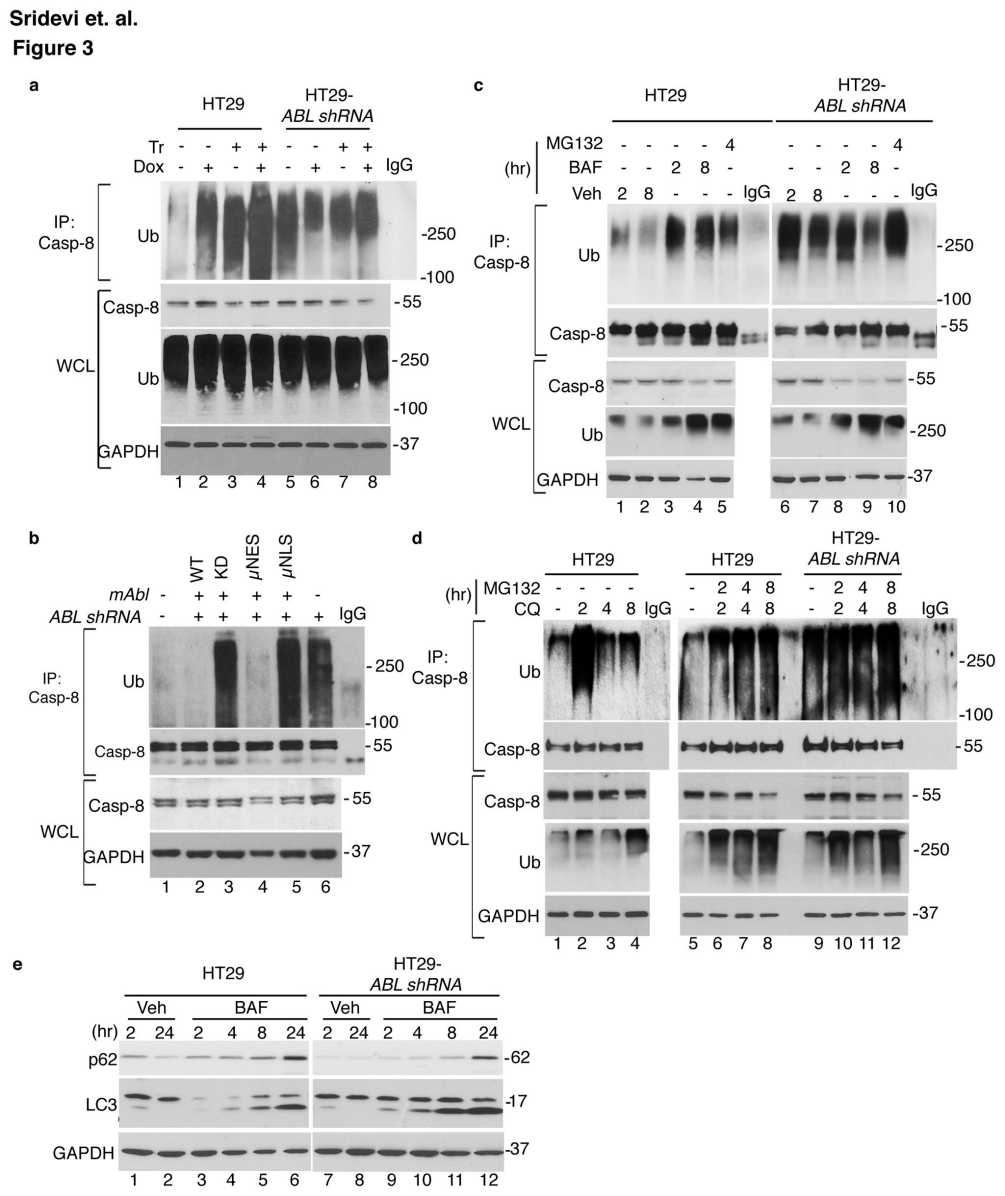
Effects of ABL-knockdown, mAbl-reconstitution and lysosomal inhibitors on ubiquitinated caspase-8. a. Inducible vs. constitutive levels of Ub-C8 in parental vs. ABL-knockdown HT29 cells. Following treatment with 2.0 µg/ml Dox and/or 200 ng/ml Tr for 6 hr, cells were lysed in 1% SDS, boiled, immunoprecipitated with anti-caspase-8 and probed with anti-Ub (see Materials & Methods). IgG: anti-caspase-8 antibody added to the lysis buffer and signals on the blot are from the antibody itself. WCL: whole cell lysates. Note that caspase-8 was mostly not ubiquitinated as the increase in Ub-C8 did not significantly reduce the intensity of the 55kd pro-caspase-8 band in WCL, and that Ub-C8 was only detected in the anti-C8 immunoprecipitates. Poly-Ub levels in WCL were not affected by the treatments with Dox and/or TRAIL or by ABL-knockdown. b. Reduction of Ub-C8 levels in ABL-knockdown cells through mAbl-reconstitution. Ub-C8 was measured as in (a). Reconstitution with WT- or µNES-mAbl reduced Ub-C8 in the ABL-knockdown cells. Reconstitution with KD or µNLS-mAbl did not reduce Ub-C8. IgG as in a. c. Effects of Bafilomycin A1 (BAF) and MG132 on Ub-C8. Cells were treated with 200 nM BAF or 20 µM MG132 for the indicated time (hr) and Ub-C8 measured as in (a). Veh: vehicle (DMSO). Note that increases in WCL poly-Ub were similar between the parental and the ABL-knockdown HT29 cells. IgG as in a. d. Effects of Chloroquine (CQ) and MG132 on Ub-C8. Cells were treated with 10 µM chloroquine (CQ) and/or 20 µM MG132 for the indicated time (hr) and Ub-C8 measured as in (a). IgG as in a. e. Effects of BAF (200 nM) on the levels of p62/SQTM1, LC3, and LC3II at the indicated time (hr). Veh: vehicle (DMSO). BAF treatment caused similar increases in p62 and LC3II in the parental and the ABL-knockdown HT29 cells.

It has been shown that TRAIL stimulates the ubiquitination of caspase-8, and this ubiquitination mechanism can either activate or degrade caspase-8 [49,50]. In HT29 cells, treatment with TRAIL or Dox caused an increase in the levels of Ub-C8, and the TRAIL+Dox effects on Ub-C8 were additive (Figure 3a). Interestingly, we observed a higher basal level of Ub-C8 in the ABL-k.d. cells, and this level was not significantly increased by TRAIL, Dox, or TRAIL+Dox (Figure 3a). Re-expression of WT- or µNES- mAbl reduced the basal levels of Ub-C8 to that of the parental HT29 cells (Figure 3b). By contrast, re-expression of KD- or µNLS- mAbl did not reduce Ub-C8 (Figures 3b). 

Because Ub-C8 can be degraded by the proteasome and the lysosome [51,52], we determined the effects of proteosomal and lysosomal inhibitors on Ub-C8 levels. In HT-29 cells, treatment with DMSO (vehicle) induced a low level of Ub-C8, which was further increased by the addition of the V-ATPase inhibitor bafilomycin A1 (BAF) (Figure 3c). Treatment with the proteosomal inhibitor MG132 also caused an increase in Ub-C8 (Figure 3c). In the ABL-k.d. cells, the higher levels of Ub-C8 were not further increased by BAF or MG132 (Figure 3c). The higher basal levels of Ub-C8 did not lead to activation of caspases in the absence of TRAIL, suggesting that the expression of Ub-C8 was not sufficient to cause caspase-8 activation in the ABL-k.d. cells (Figure 3c). Similar to BAF, inhibition of the lysosomal function by chloroquine (CQ) also induced Ub-C8 in HT-29 but not in ABL-k.d. cells (Figure 3d). In HT29 cells, CQ treatment induced a higher level of Ub-C8 at 2 hr than later time points, and co-treatment of CQ+MG132 stabilized the Ub-C8 levels (Figure 3d). In the ABL-k.d. cells, the higher Ub-C8 levels were not further increased by treatment with CQ or CQ+MG132 (Figure 3d). We found that BAF, CQ and MG132 induced comparable increases in poly-ubiquitinated proteins in the HT29 and the ABL-k.d. cells (Figure 3c, d), suggesting that ABL did not affect the degradation of Ub-proteins in general. We found similar levels of LC3 and similar accumulation of LC3-II after BAF addition in the parental and the ABL-knockdown cells (Figure 3e), suggesting that the autophagic flux was not affected by the loss of ABL function. BAF treatment also increased the levels of p62/SQSTM1 (Figure 3e), showing that lysosomal degradation of p62 was not affected by ABL knockdown. These results suggest that ubiquitination of caspase-8 could occur in the absence of TRAIL and the basal Ub-C8 levels were kept low by lysosomal and proteosomal degradation in HT-29 cells but not in the ABL-k.d. cells. 

### Chloroquine (CQ) stimulates TRAIL-induced apoptosis in cells expressing ABL or mAbl

**Figure 4:**
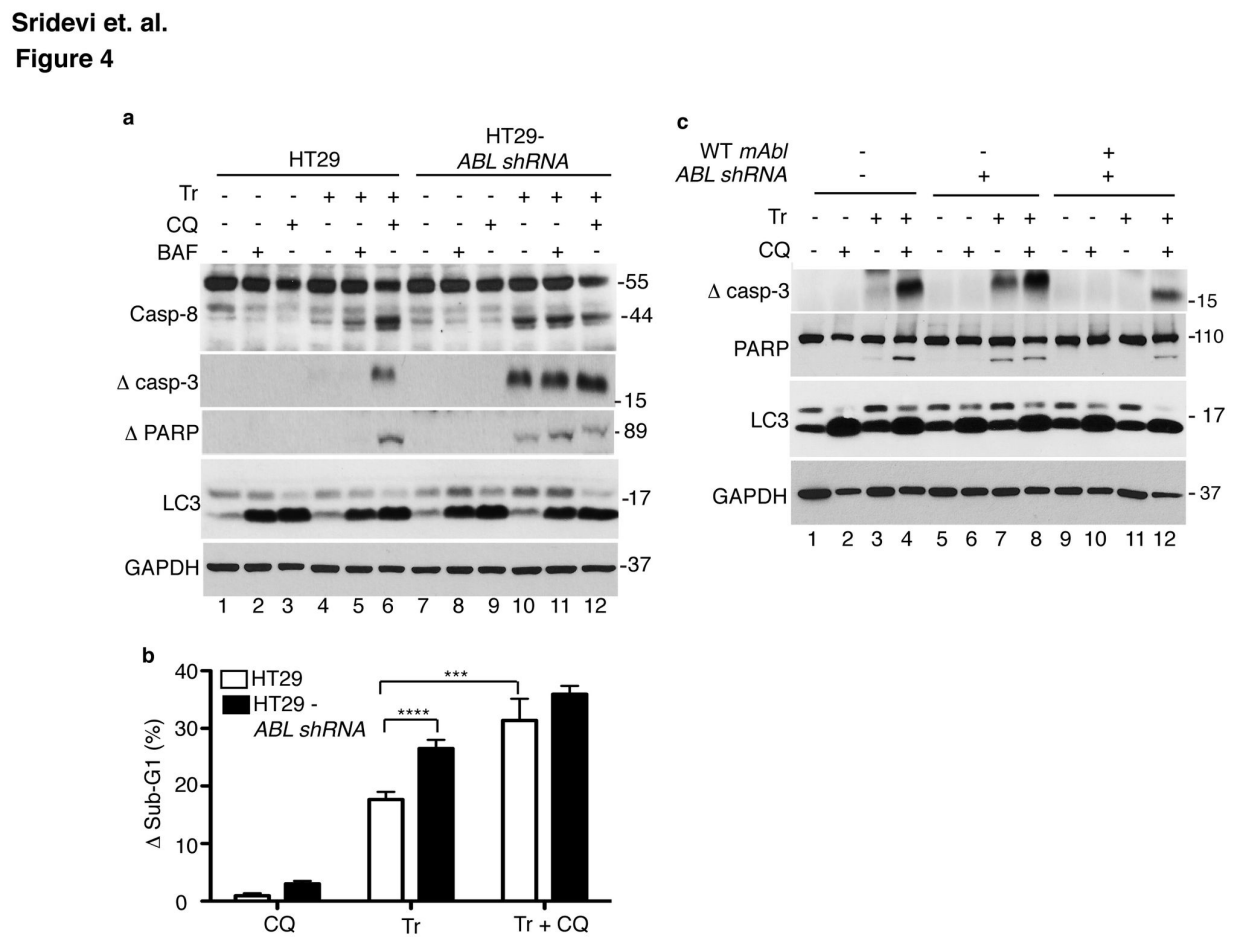
Chloroquine (CQ) stimulates TRAIL-induced apoptosis in cells expressing ABL or mAbl. a. CQ co-treatment enhanced TRAIL-induced caspase and PARP1 cleavage in parental but not ABL-knockdown HT29 cells. CQ (10 µM) or BAF (200 nM) was added 2 hr before TRAIL (Tr, 200 ng/ml, 6 hr). Whole cell lysates were probed with the indicated antibodies. Note that CQ and BAF increased LC3II levels and this was not affected by TRAIL or ABL-knockdown. b. The sub-G1 response to CQ, TRAIL and TRAIL+CQ. Cells were pre-treated with CQ (10 µM, 2 hr) before TRAIL addition (Tr, 200 ng/ml, 16 hr). The sub-G1 fractions of vehicle-treated populations (between 3 to 5 %) were subtracted from those of CQ, Tr or Tr+CQ treated populations (∆Sub-G1). Values shown are mean and standard deviation of ∆sub-G1 from 3 independent experiments. *** = p < 0.0005, **** = p < 0.00005. c. Effects of mAbl-reconstitution on CQ stimulation of TRAIL-induced caspase and PARP1 cleavage in the ABL-knockdown cells. Cells were treated as in (a) and whole cell lysates probed with the indicated antibodies.

Since the higher basal levels of Ub-C8 correlated with enhanced apoptotic response to TRAIL in the ABL-knockdown cells (Figure 3b and Figure 2f), and since lysosomal inhibition increased the basal levels of Ub-C8, we examined the effect of BAF and CQ on TRAIL-induced cleavage of caspase-8, caspase-3 and PARP1 (Figure 4a). We found that BAF or CQ alone did not stimulate those cleavages (Figure 4a). Interestingly, CQ co-treatment stimulated TRAIL-induced cleavage of caspase-8, caspase-3 and PARP1 in HT29 cells, whereas BAF did not have a similar effect (Figure 4a). With the ABL-knockdown cells, the already enhanced cleavage of caspase-8, caspase-3 and PARP1 induced by TRAIL was not further stimulated by co-treatment with CQ (Figure 4a). Both CQ and BAF caused LC3-II accumulation to similar levels in the HT-29 and the ABL-k.d., cells in the absence or presence of TRAIL (Figure 3f), indicating similar lysosomal inhibition in both cell lines. However, BAF is a specific inhibitor of the V-ATPase that acidifies the late endosomes and the lysosomes [53], whereas CQ is a lysosomotropic drug that accumulates in the acidic vesicles and organelles [54]. While both drugs inhibited the degradation of LC3-II, they might have different effects on other lysosomal functions. We found that the proteosomal inhibitor MG132 also did not stimulate TRAIL-induced caspase activation (data not shown). These results showed that lysosomal inhibition by BAF (Figure 4a) and Ub-C8 accumulation by BAF or MG132 (Figure 3c) were not sufficient to enhance TRAIL-induced caspase activation. Therefore, additional alterations caused by CQ but not by BAF or MG132 were likely to also be required for apoptosis stimulation. 

CQ treatment alone did not cause a significant increase in the sub-G1 fraction (Figure 4b). In the absence of CQ, TRAIL-induced sub-G1 fraction was significantly higher in the ABL-k.d. than the parental HT-29 cells (Figure 4b, Tr). With CQ co-treatment, TRAIL induced sub-G1 levels were significantly increased in HT-29 cells (Figure 4b, compare white bars). With TRAIL+CQ treatment, the sub-G1 levels were found to be comparable in HT-29 and ABL-k.d. cells (Figure 4b, Tr+CQ). Re-expression of mAbl in the ABL-knockdown cells suppressed TRAIL-induced caspase activation (Figures 1d, 4c) and re-sensitized cells to the caspase-activating effect of CQ (Figure 4c). These results suggest that CQ enhancement of TRAIL-induced apoptotic response was dependent on the expression ABL kinase.

### Persistent inhibition of ABL tyrosine kinase is required to enhance TRAIL-induced apoptosis

**Figure 5:**
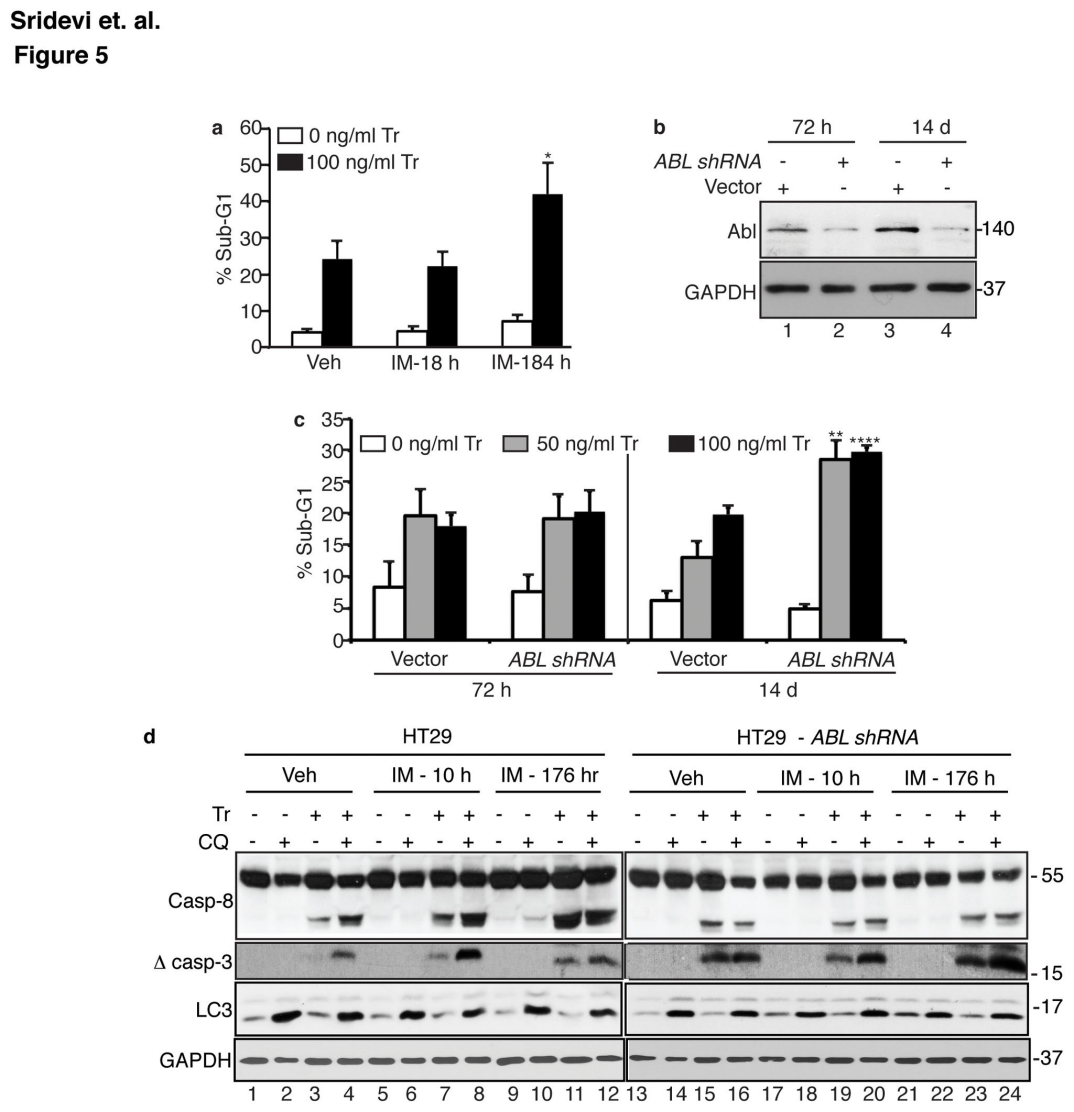
Persistent inhibition of ABL tyrosine kinase is required to enhance TRAIL-induced apoptosis. a. Short-term vs. long-term effect of imatinib on TRAIL-induced sub-G1 response. HT29 cells treated with vehicle (Veh, PBS) or imatinib (IM, 2 µM). The IM-18 hr cells were pre-treated with IM for 2 hr before adding TRAIL (Tr, 100 ng/ml, 16 hr). The IM-184 hr cells were pre-treated with IM for 7 days (168 hr) with daily addition of IM before adding TRAIL. Values shown are mean and standard deviation from 3 independent experiments. * = p < 0.05. b. Reduction in ABL levels by ABL-shRNA. Whole cell lysates at the indicated time (h: hours; d: days) after infection of HT29 cells with lentiviral particles carrying the vector or the ABL-shRNA expression cassette were . c Enhanced sub-G1 response to TRAIL was observed only with ABL-knockdown cells after 14 days of passages. Treatment with the indicated dose of TRAIL was for 16 hr. Values shown are mean and standard deviation from 3 independent experiments. ** = p < 0.005 and **** = p < 0.00005. d. Persistent treatment with IM abolished the stimulatory effect of CQ on TRAIL-induced caspase cleavage. Cells treated with IM for 2 hr (IM-10 hr) or 7-days (IM-176 hr) were then treated with CQ (10 µM, 2hr) and/or TRAIL (Tr, 200 ng/ml, 6 hr) and whole cell lysates were probed with the indicated antibodies. Long-term treatment with IM for 7-days abolished the stimulatory effect of CQ in HT29 cells. Neither the short-term nor the long-term IM treatment affected TRAIL-induced caspase activation in the ABL-knockdown cells, showing that the IM effect was due to ABL kinase inhibition.

Because the ABL kinase activity is required to suppress TRAIL-induced apoptosis (Figure 2f), we expected that the ABL kinase inhibitors such as imatinib (IM) would enhance TRAIL-induced apoptotic response. However, we found that addition of imatinib at 2 hr. prior to TRAIL addition and throughout the TRAIL treatment period (for a total of 18 hr.) did not affect the sub-G1 response (Figure 5a, Figure S3a). Similarly negative results were obtained with two other ABL kinase inhibitors, dasatinib (DA) and nilotinib (NI) (Figure S3b). This discrepancy between the KD-mAbl and the IM effects on apoptosis could be due to the duration of ABL kinase inhibition. The KD-mAbl cells were established by retroviral infection of the ABL-knockdown cells, and their response to TRAIL was examined after 10-20 days in culture. To determine if prolonged inhibition of ABL kinase is required to enhance apoptosis, we pre-treated HT29 cells with IM daily for 7 days prior to TRAIL addition and throughout the TRAIL treatment period (184 hr. total). We found that pre-treatment of HT29 cells with IM for 7 days was able to cause an enhanced sub-G1 response to TRAIL (Figure 5b). 

To determine if the ABL-knockdown effect on apoptosis also required the persistent loss of its kinase function, we examined TRAIL-induced sub-G1 response in HT29 cells at 72 hr. or 14 days after infection with the ABL-shRNA lentivirus. When compared to vector-infected cells, ABL-shRNA-Infected cells showed comparable ABL reduction at 72 hr. and 14 days (Figure 5b). However, the knockdown of ABL for 72 hr. did not enhance the sub-G1 response to TRAIL, whereas the knockdown of ABL for 14 days enhanced the apoptotic response (Figure 5c). These results show that the persistent loss of ABL kinase function is required for cells to become sensitized to TRAIL-induced apoptosis. 

We then compared the effects of short-term vs. long-term IM treatment on the CQ-response in HT29 cells (Figure 5d). CQ-induced accumulation of LC3II was not affected by IM short-term or long-term treatments in the parental or the ABL-k.d. cells (Figure 5d). In HT29 parental cells, the CQ-mediated stimulation of TRAIL-induced cleavage of caspase-8 and caspase-3 was not disrupted by the short-term treatment with IM (Figure 5d, compare lanes 3, 4 to lanes 7,8). However, in HT29 cells pretreated with IM for 7 days, CQ did not further stimulate the already enhanced cleavage of caspases induced by TRAIL (Figure 5e, compare lanes 3, 4 to lanes 11, 12). Neither the short-term nor the long-term treatment with IM affected TRAIL-induced caspase cleavage in the ABL-k.d. cells (Figure 5d, compare lanes 15, 19, 23), confirming that the IM effect was mediated through the inhibition of ABL. Similarly, CQ did not stimulate TRAIL-induced caspase cleavage in the ABL-k.d. cells treated with IM for 18 hr. or 184 hr. (Figure 5d, compare lanes 16, 20, 24). Together, these results show that the persistent long-term treatment with IM was required to enhance the apoptotic response to TRAIL and to abolish the pro-apoptotic effect of CQ. 

## Discussion and Conclusions

The ubiquitously expressed ABL tyrosine kinase is activated by a variety of signals including growth factors, cytokines, antigens, cell adhesion, and bacterial infection [15,31,33-37]. The ABL tyrosine kinase is also activated by DNA damage and oxidative stress [16,18,22,38,55]. In the cytoplasm, activated ABL tyrosine kinase phosphorylates many different proteins to regulate a wide range of biological processes including F-actin polymerization, receptor endocytosis, and PARKIN-dependent mitophagy [56,57]. In the nucleus, activated ABL tyrosine kinase phosphorylates transcription factors and chromatin modifying enzymes to regulate gene expression [38,58,59]. In DNA damage response (DDR), activated ATM stimulates ABL phosphorylation and nuclear accumulation to activate the p53-family of transcription factors, leading to the induction of intrinsic apoptosis [16,17,55]. Recently, it was demonstrated that cisplatin-induced apoptosis of renal proximal tubule cells is defective in mice expressing the nuclear import-defective Abl-µNLS protein, providing in vivo evidence for the pro-apoptotic function of nuclear ABL tyrosine kinase in DDR [19]. Taken together, the studies of ABL have shown that this ubiquitously expressed tyrosine kinase is involved in many different signaling pathways and that its biological effects are determined by the activating signals and by the subcellular location of the activated ABL kinase.

A previous study has shown that the ABL tyrosine kinase is activated by TRAIL to stimulate JNK and apoptosis [25]. TRAIL induces apoptosis by activating its death receptor and the assembly of the death-inducing signaling complex (DISC) consisting of receptor, FADD and caspase-8 [60]. There is no evidence that the ABL kinase is recruited to the DISC to stimulate caspase-8 activation. However, there are many reports showing that the ABL-JNK-apoptosis pathway is activated by DNA damage [32,61,62]. Interestingly, TRAIL has been shown to activate the DDR through caspase-dependent DNA fragmentation [23,24]. It is likely that TRAIL can stimulate the ABL-JNK-apoptosis pathway downstream of caspase activation through the DDR. This interpretation is consistent with published results showing that the pro-apoptotic effectors of the DDR, such as CHK2, ABL, p73 and JNK, can contribute to TRAIL-induced apoptosis [15,24,25]. 

In this study, we show that the nuclear ABL tyrosine kinase also has an anti-apoptotic effect because the persistent disruption of nuclear ABL kinase function can cause cells to become more sensitive to TRAIL-induced caspase-8 activation. In other words, nuclear ABL tyrosine kinase has an anti-apoptotic effect that acts upstream of caspase-8 activation. Unlike its pro-apoptotic function, this anti-apoptotic ABL effect is not immediately lost upon its knockdown or the inhibition of its kinase function. To enhance TRAIL-induced caspase-8 activation requires stable knockdown or persistent kinase inhibition for several days. These findings suggest that the nuclear ABL kinase may be required to maintain the expression of one or more long-lived cellular components that can inhibit TRAIL-induced apoptosis. These inhibitors decay slowly after the loss of ABL function; as a result, enhanced apoptosis is only observed several days after ABL-knockdown or kinase inhibition. Previous studies have identified a number of transcription regulators that are phosphorylated by the ABL tyrosine kinase, including the C-terminal repeated domain (CTD) of RNA polymerase II [63–65], p53 [66], p73 [16], p63 [39], MDM2 [67], MDMX [68], and others [21]. It is therefore possible that nuclear ABL can regulate the expression of long-lived inhibitors of TRAIL-induced apoptosis. Alternatively, the persistent loss of nuclear ABL kinase function may induce adaptive responses that result in the enhanced caspase-8 activation by TRAIL. 

It has been shown that TRAIL can stimulate Ub-C8 that contributes to caspase activation or degradation [49,50]. It has also been shown that Ub-C8 binds p62/SQSTM1 for delivery to the autophagosome and the autolysosome [51,52]. We have found that treatment with proteosomal or lysosomal inhibitors could induce Ub-C8, suggesting that caspase-8 can be ubiquitinated without TRAIL stimulation and the basal Ub-C8 is kept low by lysosomal and proteosomal degradation. We show that the basal levels of Ub-C8 were higher in the ABL-knockdown cells and that reconstitution of the wild type, but not the nuclear import- or kinase-defective Abl, reduced the basal Ub-C8. The basal accumulation of Ub-C8 is clearly not sufficient for its activation. However, the disruption of Ub-C8 degradation in the ABL-knockdown cells could be a contributing factor in the enhanced caspase-8 activation by TRAIL.

It has been shown that the lysosomal degradative function could be reduced by imatinib treatment or by the combined knockdown of ABL and its related kinase ARG [69]. A reduction in lysosomal function can explain the accumulation of Ub-C8 in the ABL-knockdown cells; however, this mechanism cannot explain the observation that LC3-II, p62/SQSTM1, or other poly-ubiquitinated proteins accumulated to similar extents in the parental and the ABL-knockdown cells following lysosomal inhibition. While the accumulation of Ub-C8 correlated with apoptosis sensitivity, the levels of Ub-C8 per se were not the determining factor of the apoptotic response. We have found that treatment with bafilomycin A1 or imatinib also caused Ub-C8 accumulation within hours (Figure 3c and data not shown), however, these immediate increases in Ub-C8 did not enhance TRAIL-induced apoptosis. Instead, treatment with chloroquine (CQ), another lysosomal inhibitor, caused the accumulation of Ub-C8 and stimulated TRAIL-induced apoptosis. CQ inhibits lysosomal degradation by accumulating in the lysosomes [54] whereas bafilomycin A1 inhibits the V-ATPase on the lysosomal membrane [53]. These two drugs may therefore affect the lysosomal function in different ways. It is interesting to find that CQ does not stimulate the already enhanced apoptotic response in the ABL-knockdown cells. We also show that short-term pre-treatment with imatinib (2 hours to 2 days) cannot enhance TRAIL-induced apoptosis, nor can it abolish the pro-apoptotic effect of CQ. However, the persistent treatment with imatinib (7 days) can enhance apoptosis and abolish the pro-apoptotic effect of CQ. Taken together, these results suggest that the nuclear ABL tyrosine kinase is required to maintain an anti-apoptotic mechanism that involves the lysosomes and is neutralized by CQ. 

The FDA-approved ABL kinase inhibitors – IM, DA and NI, have been used to treat CML patients with continuous daily dosing for many years [40,42,43]. However, the side effects associated with long-term administration of these kinase inhibitors in patients have not been studied. Our finding that persistent treatment with IM, DA or NI can disrupt nuclear ABL tyrosine kinase function to enhance TRAIL-induced apoptosis may have clinical ramifications. It would be of interest to determine if long-term treatments with these kinase inhibitors can enhance caspase-8 activation by other inducers. It would also be of interest to determine if a pro-longed pre-treatment with these drugs can be used to enhance the efficacy of TRAIL-based cancer therapy. 

## Supporting Information

Figure S1 **Knockdown of ABL sensitized cancer and non-cancer cells to TRAIL.**
(TIF)

Figure S2 **Levels of pro-apoptotic and anti-apoptotic proteins in parental and ABL knockdown HT29 cells.**
(TIF)

Figure S3 **Short-term inhibition of ABL tyrosine kinase with Imatinib (IM), Dasatinib (DA) and Nilotinib (NI) does not affect the Sub-G1 response to TRAIL.**
(TIF)
