# Supplementary figures and images for: Persistent Inhibition of ABL Tyrosine Kinase Causes Enhanced Apoptotic Response to TRAIL and Disrupts the Pro-Apoptotic Effect of Chloroquine
Source: PLoS One. 2013 Oct 11;8(10):e77495. doi: 10.1371/journal.pone.0077495 (PMC3795698; doi:10.1371/journal.pone.0077495)

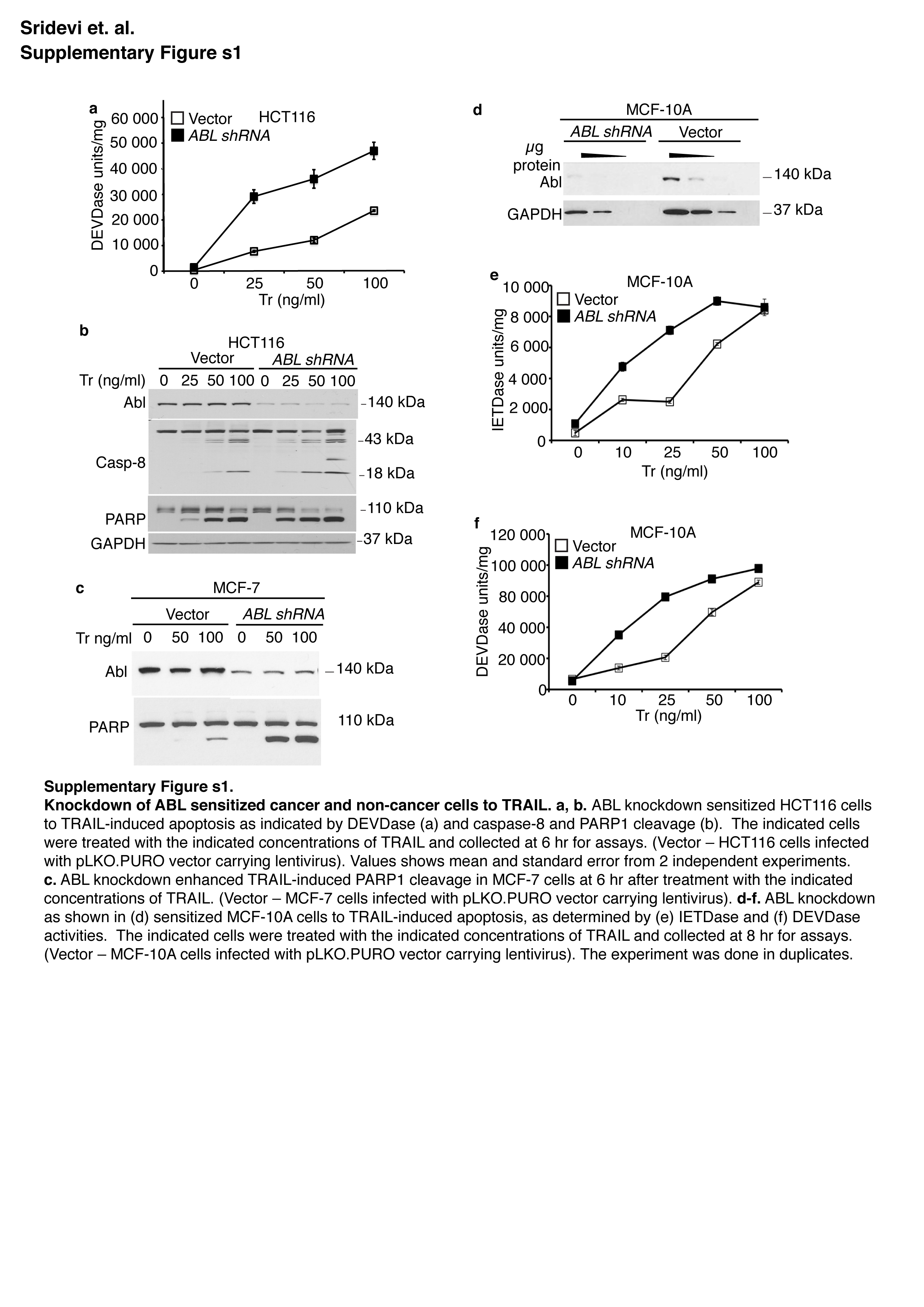

Supplement: Figure S1 — Knockdown of ABL sensitized cancer and non-cancer cells to TRAIL. (TIF) [file pone.0077495.s001.tif]

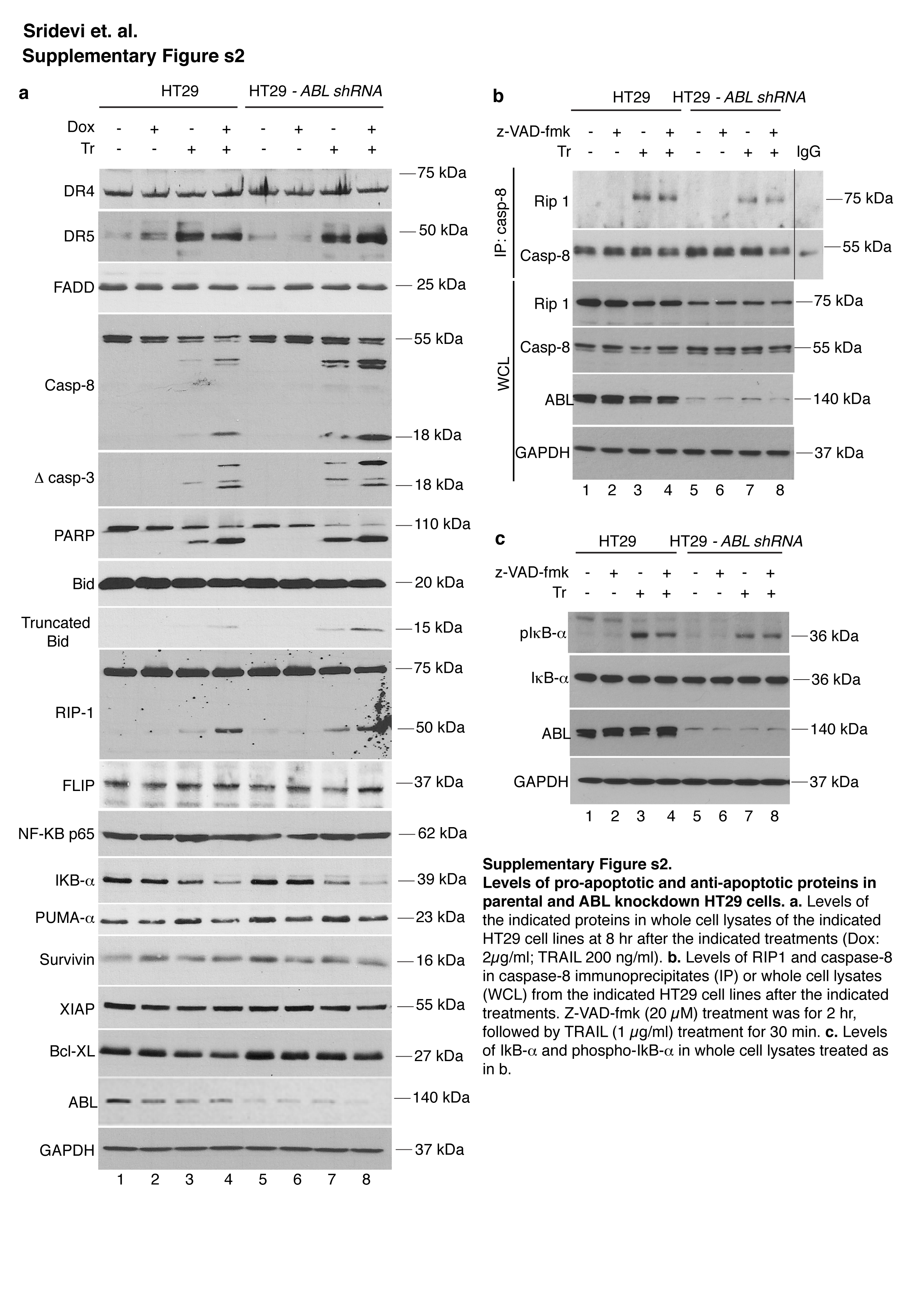

Supplement: Figure S2 — Levels of pro-apoptotic and anti-apoptotic proteins in parental and ABL knockdown HT29 cells. (TIF) [file pone.0077495.s002.tif]

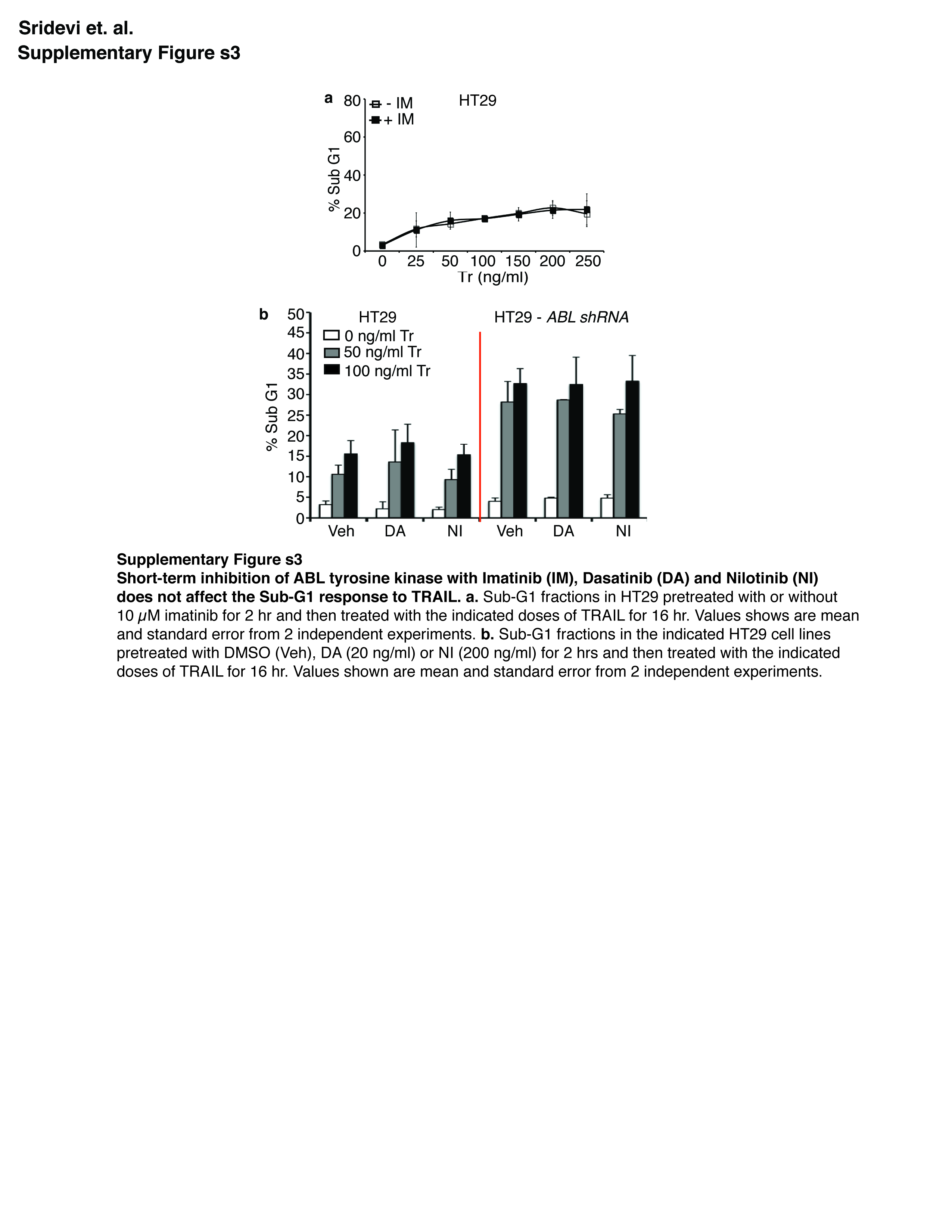

Supplement: Figure S3 — Short-term inhibition of ABL tyrosine kinase with Imatinib (IM), Dasatinib (DA) and Nilotinib (NI) does not affect the Sub-G1 response to TRAIL. (TIF) [file pone.0077495.s003.tif]
